# COVID-19-Associated Thrombotic Complication: Is It Pulmonary Embolism or In Situ Thrombosis?

**DOI:** 10.1155/2023/3844069

**Published:** 2023-07-03

**Authors:** Rashid AL Umairi, Khadija AL Adawi, Maryam AL Khoori, Ahmed AL Lawati, Sachin Jose

**Affiliations:** ^1^Radiology Department, The Royal Hospital, Muscat, Oman; ^2^Medical Specialty Board, Muscat, Oman

## Abstract

**Objectives:**

Acute pulmonary embolism is a protentional fatal complication of COVID-19. The aim of this study is to investigate whether pulmonary embolism is due to thrombus migration from the venous circulation to the pulmonary arteries or due to local thrombus formation secondary to local inflammation. This was determined by looking at the distribution of pulmonary embolism in relation to lung parenchymal changes in patients with COVID-19 pneumonia.

**Methods:**

Retrospectively, we identified pulmonary computed tomography angiography (CTPA) of patients admitted to the Royal Hospital between November 1st, 2020, and October 31, 2021, with a confirmed diagnosis of COVID-19. The CTPAs were examined for the presence of pulmonary embolism and the distribution of the pulmonary embolism in relation with lung parenchymal changes.

**Results:**

A total of 215 patients admitted with COVID-19 pneumonia had CTPA. Out of them, 64 patients had pulmonary embolisms (45 men and 19 women; mean age: 58.4 years with a range of 36–98 years). The prevalence of pulmonary embolism (PE) was 29.8% (64/215). Pulmonary embolism was more frequently seen in the lower lobes. 51 patients had PE within the diseased lung parenchyma and 13 patients had PE within normal lung parenchyma.

**Conclusion:**

The strong association between pulmonary artery embolism and lung parenchymal changes in patients admitted with COVID-19 pneumonia suggests local thrombus formation.

## 1. Introduction

Thrombotic complications including disseminated intravascular coagulation, deep vein thrombosis, and pulmonary embolisms are common complications among patients admitted with COVID-19 pneumonia [1–3]. However, the pathophysiology behind the increased incidence of thrombotic complications among patients with COVID-19 pneumonia is unclear. It has been suggested that the endothelial inflammation and damage caused by COVID-19 infection can lead to local thrombus formation mediated by the activation of complement pathways and associated procoagulant state [4]. This would mean that pulmonary embolism is formed locally in the lung parenchyma in patients affected with COVID-19 pneumonia. As per the author's knowledge, only one study with a very limited number of patients has looked at the distribution of PE in relation with the lung parenchymal changes [5]. Therefore, the aim of our study was to investigate if pulmonary embolism is due to thrombus migration from the venous circulation to the pulmonary arteries or due to local thrombus formation secondary to local inflammation.

## 2. Methods

We conducted a retrospective study to identify all computed tomography pulmonary angiography (CTPA) scans performed for patients admitted to our tertiary hospital with a confirmed diagnosis of COVID-19 infection, between November 1st, 2020, and October 31, 2021.

The diagnosis of COVID-19 was based on a laboratory-confirmed positive RT-PCR for SARS-CoV-2 obtained from the nasopharynx and oropharynx of all patients. Medical records of included patients were reviewed and demographic characteristics, vital signs, presenting symptoms, comorbidities, and D-dimer were documented.

The computed tomography pulmonary angiography (CTPA) was performed using a dual source 256 slice (2 × 128) scanner (SOMATOM Definition Flash, Siemens AG), with a rotation time of 280 minutes. All scans started with a tomogram followed by a contrast-enhanced scan of the pulmonary arteries with a slice thickness of 1 mm which was acquired during breath holding after inspiration or free breathing if the patient was not able to follow the breathing instruction. CTPAs were initially reported by the attending radiologist for the presence of pulmonary embolism (PE). Then, they were again analyzed by 4 board-certified radiologists for the distribution of PEs in relation with lung parenchymal changes. All CTPAs were reviewed using a dedicated radiology PACS system. Lungs were divided into 18 segments and each segment was evaluated for parenchymal changes and for the presence of pulmonary embolism. Pulmonary embolism was called in situ if it was within a segment with lung changes, and not in situ if the segment had no parenchymal changes.

The ethical approval was obtained from the scientific research committee at our institute. Informed consents were waived.

## 3. Statistics

Continuous variables were presented as mean, median, and standard deviation whereas, categorical variables were presented as frequency and percentage. The comparison of means between the two groups was assessed using the independent samples *t*-test or the Mann−Whitney *U* test, as appropriate. The association between two categorical variables was assessed using an appropriate chi-square test (likelihood ratio test or Fisher's exact test). A *P* value of less than 0.05 was considered statistically significant. All analysis was carried out using the IBM SPSS statistics version 26.0.

## 4. Results

Over a one-year period, we identified 215 patients admitted with COVID-19 pneumonia and had CTPA. Out of the 215 patients, 64 (29.8%, 95% CI = 23.7%–36.4%) of them had positive CTPA for pulmonary embolism (45 men and 19 women; mean age, 58.4 years with range 36–98 years) and they were included in the statistical analysis. The demographical and clinical features of the included patients are summarized in Table 1.

The analysis of the distribution of pulmonary embolism on the lung lobe level showed that 51 (79.7%) patients had PE within the diseased lung parenchyma (in situ) and 13(20.3%) patients had PE within the normal lung parenchyma (not in situ) (Table 1). There was a predilection for right lower lobe involvement (71.9%) followed by left lower lobe (59.4%) with a statistically significant difference (Tables 2–4).

## 5. Discussion

Coagulopathy, including pulmonary embolism (PE), is a common complication among patients admitted with COVID-19 infection, and it is usually associated with a poor prognosis [1, 6]. In this study, the prevalence of PE among the patients admitted with COVID-19 infection who had PCTA was 29.8%. In the literature, the incidence of PE among patients admitted with COVID-19 pneumonia is variable. In a systematic review and meta-analysis by Liu et al. [7], the reported overall incidence of PE was 17.6% (95% CI: 12.3%–23.5%) and 21.7% (95% CI: 14.8%–29.3%) in the patients with a severe disease.

The exact mechanism behind the increased thrombotic complications among patients with COVID-19 infection is not clear. However, there are several potential mechanisms that might be responsible for promoting the risk of coagulopathy such as the severe inflammatory response and disseminated intravascular coagulation [8], blood vessel endothelial damage caused directly by the virus and the associated local inflammatory process [9], drug-drug reactions, and the limited mobility among patients with COVID-19 pneumonia admitted to the intensive care unit (ICU) [8].

The standard management of patients admitted with COVID-19 pneumonia and those who were found to have PE is therapeutic doses of anticoagulation. Different agents can be used including unfractionated heparin, low-molecular-weight heparin (LMWH), and direct oral anticoagulant [8]. If we consider that platelets play an important role in local pulmonary thrombosis compared with pulmonary embolism, then it might be necessary to treat these patients with antiplatelet agents along with anticoagulation agents. The latter works mainly on pulmonary embolism, whereas antiplatelet agents, such as aspirin and clopidogrel, block antiplatelet activation [10–12]. The hypothesis of our study is that PE is due to local clot formation secondary to local inflammation and damage caused directly by the virus. Mueller−Peltzer et al. have shown that PE is more frequently encountered in the opacified regions of the lungs and concluded that this might be due to a local clot formation [5]. However, the population in their study was small. In our study, we included 215 patients admitted at our institute with a confirmed diagnosis of COVID-19 pneumonia between November 1st, 2020, and October 31, 2021, and out of them 64 patients had PE, 23.7% of patients had PE within the diseased lung parenchyma (in situ), and 6% of patients had PE within the normal lung parenchyma (not in situ). This shows a strong association between PE and lung parenchymal changes suggesting that PE is locally formed. Local thrombosis in the pulmonary artery is likely due to the strong inflammatory process that results in pulmonary artery endothelial damage. In addition, SARS-CoV-2 might activate the coagulation pathway by binding the ACE-2 receptor of type II pneumocytes and then dysregulating the kallikrein/kinin system [13]. If COVID-19 is associated with local thrombus formation rather than PE, then the management of coagulopathy in patients with COVID-19 infection might need to be adjusted by adding antiplatelets along with LMWH. In addition, the commonly used Wells pretest probability score may not be valid since it depends on the presence/absence of clinical signs of deep vein thrombosis (DVT) [14], which would not be relevant in local pulmonary artery thrombosis.

## 6. Limitation

This study has a few limitations. First, this study is a retrospective study with a relatively small sample size, and still, data need to be confirmed in a larger population to prove a local clot formation. Second, most of our patients were ventilated and this can result in a motion artifact during the scan that limits the assessment of the pulmonary arteries. Third, we cannot exclude deep vein thrombosis in our population as Doppler ultrasound of the lower limbs was not routinely performed.

## 7. Conclusion

Pulmonary embolism is a relatively common complication among patients admitted with COVID-19 pneumonia. A high frequency of PE in the diseased lungs might suggest that PE in patients with COVID-19 pneumonia is due to local thrombus formation. This requires specific assessment and an appropriate therapeutic response.

## Figures and Tables

**Table 1:** Clinical features of patients with pulmonary embolism (PE) in situ and PE not in situ.

Variable	PE in situ (*n* = 51)	PE not in situ (*n* = 13)	*P* value
	*n* (%)	*n* (%)	
*Sex*			
Male	32 (64.0)	12 (92.3)	0.086
Female	18 (36.0)	1 (7.7)	
Age, mean ± SD	58.70 ± 12.73	55.0 ± 14.38	0.367
*Distribution*			
Central	1 (2.0)	0 (0)	0.794
Peripheral	35 (68.6)	9 (69.2)	
Both	15 (29.4)	4 (30.8)	
COVID-19			
Active COVID-19	41 (80.4)	9 (69.2)	0.457
Post COVID-19	10 (19.6)	4 (30.8)	
Superadded bacterial infection	5 (9.8)	0 (0)	0.574
Thrombosis elsewhere	2 (3.9)	2 (15.4)	0.181
Fever	18 (35.3)	4 (30.8)	1.000
Runny nose	2 (3.9)	1 (7.7)	0.500
Cough	21 (41.2)	4 (30.8)	0.544
SOB	35 (68.6)	12 (92.3)	0.157
Headache	1 (2.0)	0 (0)	1.000
Vomiting	1 (2.0)	0 (0)	1.000
Muscle ache/body ache	4 (7.8)	1 (7.7)	1.000
Malaise	2 (3.9)	1 (7.7)	0.500
Lethargy	3 (5.9)	0 (0)	1.000
Pharyngeal discomfort/pain	1 (2.0)	0 (0)	1.000
Chest pain	3 (5.9)	2 (15.4)	0.266
Pleuritic chest pain	1 (2.0)	0 (0)	1.000
Abdominal pain	1 (2.0)	1 (7.7)	0.368
Diarrhea	1 (2.0)	1 (7.7)	0.368
Hemoptysis	1 (2.0)	1 (7.7)	0.368
SYNCOPE	1 (2.0)	0 (0)	1.000
Anorexia	2 (3.9)	0 (0)	1.000
*eGFR*			
Normal	25 (54.3)	8 (61.5)	0.757
Abnormal	21 (45.7)	5 (38.5)	
*CRP*			
Normal	6 (20.0)	3 (37.5)	0.363
Abnormal	24 (80.0)	5 (62.5)	
*D-dimer*			
Normal	1 (7.1)	2 (33.3)	0.202
Abnormal	13 (92.9)	4 (66.7)	
*Platelets*			
Normal	33 (71.7)	10 (83.3)	0.712
Abnormal	13 (28.3)	2 (16.7)	
DM	22 (43.1)	3 (23.1)	0.220
IHD	7 (13.7)	0 (0)	0.328
DLP	7 (13.7)	2 (15.4)	1.000
CKD	6 (11.8)	1 (7.7)	1.000
Obesity	2 (3.9)	2 (15.4)	0.181
Cirrhosis	4 (7.8)	0 (0)	0.574
Asthma/ILD/COPD/bronchiectasis	2 (3.9)	1 (7.7)	0.500
Smoking	4 (7.8)	0 (0)	0.574
HTN	16 (31.4)	3 (23.1)	0.739
Cancer	1 (2.0)	0 (0)	1.000
ICU	7 (13.7)	2 (15.4)	1.000
Intubation	5 (9.8)	2 (15.4)	0.623
Expired	18 (35.3)	3 (23.1)	0.518

**Table 2:** Distribution of pulmonary embolism (PE).

Variable	PE in situ (*n* = 51)
	*n* (%)
RUL-apical	11 (17.2)
RUL-anterior	15 (23.4)
RUL-posterior	11 (17.2)
RML-medial	10 (15.6)
RML-lateral	11 (17.2)
RLL-superior	17 (26.6)
RLL-medial	13 (20.3)
RLL-lateral	22 (34.4)
RLL-posterior	34 (53.1)
RLL-anterior	10 (15.6)
LUL-apicoposterior	8 (12.5)
LUL-anterior	7 (10.9)
Lingula-superior	6 (9.4)
Lingula-inferior	6 (9.4)
LLL-superior	7 (10.9)
LLL-lateral	11 (17.2)
LLL-anteromedial	12 (18.8)
LLL-posterior	17 (26.6)

**Table 3:** Distribution of pulmonary embolism (PE) by lung lobes.

Lung lobes	Number of pulmonary embolism (PE)
RUL	77
RML	36
RLL	150
LUL	36
Lingula	21
LLL	77

**Table 4:** Association between pulmonary embolism (PE) and lung lobes.

Lung lobes	Pulmonary embolism (PE)		*P* value
	No	Yes	
	*n* (%)	*n* (%)	
RUL	31 (48.4)	33 (51.6)	<0.001
RML	39 (60.9)	25 (39.1)	
RLL	18 (28.1)	46 (71.9)	
LUL	41 (64.1)	23 (35.9)	
Lingula	50 (78.1)	14 (21.9)	
LLL	26 (40.6)	38 (59.4)	

## Data Availability

The data used to support the findings of this study are available from the corresponding author upon request.
